# Cardiometabolic Effects of Cabergoline and Combined Oral Contraceptive Pills in Young Women with Hyperprolactinemia: A Pilot Study

**DOI:** 10.3390/jcm12093208

**Published:** 2023-04-29

**Authors:** Robert Krysiak, Karolina Kowalcze, Bogusław Okopień

**Affiliations:** 1 Department of Internal Medicine and Clinical Pharmacology, Medical University of Silesia, Medyków 18, 40-752 Katowice, Poland; bokopien@sum.edu.pl; 2 Department of Pediatrics in Bytom, School of Health Sciences in Katowice, Medical University of Silesia, Stefana Batorego 15, 41-902 Bytom, Poland; kkowalcze@sum.edu.pl

**Keywords:** cardiometabolic risk, dopamine agonists, oral combined contraceptive pills, prolactin excess

## Abstract

Although dopaminergic agents are the drugs of choice in treatment of prolactin excess, women who cannot be treated with these agents are recommended to receive estrogen preparations. The aim of this study was to compare cardiometabolic effects of both treatment options. The study population included three groups of young women. Subjects with mild-to-moderate hyperprolactinemia received either low-dose cabergoline or oral combined contraceptives (ethinyl estradiol plus desogestrel), while normoprolactinemic women were drug-naive. Plasma prolactin, glucose homeostasis markers, lipids, circulating levels of uric acid, high-sensitivity C-reactive protein (hsCRP), fibrinogen and homocysteine, and the urinary albumin-to-creatinine ratio (UACR) were assessed at entry and six months later. Hyperprolactinemic women differed from normoprolactinemic ones in glucose homeostasis markers, high-density lipoprotein (HDL)-cholesterol, triglycerides, uric acid, hsCRP, fibrinogen, homocysteine and UACR. Cabergoline decreased total and monomeric prolactin levels, which was accompanied by normalization of glucose, insulin sensitivity, glycated hemoglobin, HDL-cholesterol, triglycerides, uric acid, hsCRP, fibrinogen, homocysteine and UACR. Despite a neutral effect on prolactin levels, combined contraceptives worsened insulin sensitivity and increased triglycerides, hsCRP, fibrinogen and UACR. At follow-up, cabergoline-treated women were characterized by a better cardiometabolic profile than women receiving ethinyl estradiol plus desogestrel. Our findings suggest that only cabergoline reduces cardiometabolic risk in young women with hyperprolactinemia.

## 1. Introduction

Apart from oligo- or amenorrhea, galactorrhea, impaired fertility and sexual disturbances [1], chronic prolactin excess is associated with increased risk of cardiovascular and metabolic complications. Patients with prolactin-secreting tumors were characterized by lower flow-mediated dilatation of a brachial artery and increased carotid-intima media thickness in comparison with healthy volunteers, and both these parameters correlated with a degree of prolactin excess [2,3]. Hyperprolactinemia was associated with increased levels of high-sensitivity C-reactive protein (hsCRP), an established marker of low-grade inflammation [3,4], while prolactin receptors were detected on the surface of macrophages of the atherosclerotic plaque at sites of most prominent inflammation [5]. Therefore, it seems that prolactin signaling contributes to the local inflammatory response within the atherosclerotic plaque and to atherogenesis [5]. Beyond differences in plasma lipids, patients with prolactinoma were characterized by increased platelet count, increased levels of fibrinogen, antithrombin III and plasminogen activator inhibitor-1, the increased plasminogen activator inhibitor-1/tissue plasminogen activator ratio and by lower concentrations of tissue factor pathway inhibitor, indicating that hyperprolactinemia may be complicated by hypercoagulability and hypofibrinolysis [6]. Many studies showed a relationship between elevated prolactin levels and the resistance of peripheral tissues to insulin, obesity/overweight, atherogenic dyslipidemia and metabolic syndrome [7,8]. Patients with essential hypertension had higher prolactin levels than matched normotensive controls [9]. Individuals with acute coronary syndromes showed significantly higher prolactin concentrations than patients with stable angina pectoris, and the maximum values were observed in patients with acute myocardial infarction [10]. Lastly, increased prolactin levels seem to be a risk factor for ischemic stroke and venous thromboembolism, and this association is partially related to a prolactin-induced increase in platelet activation [11,12].

Adverse metabolic disturbances and proatherosclerotic effects may be reversed or alleviated by dopaminergic agents, the drugs of choice in the treatment of prolactin excess [13]. In numerous studies, dopamine agonists reduced glucose levels, improved insulin sensitivity, decreased glycated hemoglobin, improved plasma lipids, reduced the body mass index (BMI), decreased waist circumference and decreased visceral adiposity [14,15,16,17,18,19]. They were also found to improve flow-mediated dilatation [4], to reduce carotid intima-media thickness [20], to decrease low-grade systemic inflammation [4], and to reduce blood pressure [9]. In turn, the increase in prolactin levels after cessation of cabergoline treatment in patients with prolactinoma was accompanied by an increase in fibrinogen levels [21].

Patients who are intolerant or having contraindications to dopamine agonists cannot be, however, treated with these agents. Women with symptomatic hyperprolactinemia who are not desirous of pregnancy are recommended to receive combined contraceptives in order to prevent the consequences of ovarian hypofunction [22]. However, combined oral contraceptive pills may increase blood pressure [23]. Moreover, these agents confer increased risk of venous thromboembolism and arterial thrombosis [24], as well as were found to deteriorate insulin sensitivity [25]. To the best of our knowledge, no previous study assessed cardiovascular and metabolic effects of combined hormonal contraceptives in women with elevated prolactin levels. Similarly, no study has compared head to head the impact of dopaminergic agents and oral contraceptive pills. The paucity of data encouraged us to compare cardiometabolic effects of cabergoline and ethinyl estradiol/desogestrel combination therapy in young women with hyperprolactinemia.

## 2. Materials and Methods

This single-center, prospective, matched cohort study was conducted between July 2018 and February 2020, and was prematurely stopped because of the outbreak of the COVID-19 pandemic. Because of the six-month interval between baseline and follow-up evaluation, our statistical analysis included only data of enrolled between July 2018 and August 2019. The study was performed in accordance with the ethical standards as laid down in the 1964 Declaration of Helsinki and its later amendments. The study protocol was approved by the local ethics committee and all participants signed informed consent after careful explanation of the study procedures. The paper was prepared in accordance with the Enhancing the Quality and Transparency of Health Research (EQUATOR) Network guidelines for observational studies (STROBE). Because the study was non-randomized and the assignment of the medical intervention was not at the discretion of the investigators (depended on treatment aims), it did not meet the criteria of a clinical trial and had not been prospectively registered in a public database.

### 2.1. Study Population

The participants of the main study were selected among young women (aged 20–50 years) with untreated hyperprolactinemia, who had been initially supervised by local healthcare providers cooperating with our research group. To be admitted to the study, the patients were required to have prolactin levels on two different occasions, four to six weeks apart, in the range between 30 and 75 ng/mL (mild-to-moderate hyperprolactinemia) and to experience symptoms of prolactin excess: amenorrhea, oligomenorrhea, galactorrhea, vaginal dryness, dyspareunia, decreased libido or infertility. The participants were divided into three study groups. Group A included 31 women desiring to become pregnant, accepting unplanned pregnancy, using non-hormonal contraception or sexually-inactive. Group B enrolled 32 sexually-active women wanting to avoid pregnancy and interested in hormonal contraception. Both groups of subjects with hyperprolactinemia were compared with 29 women in whom prolactin levels were within the reference range, serving as a control group (group C). The enrolled patients were selected from larger populations of potential participants (118 with hyperprolactinemia and 52 with normal prolactin levels) in order to obtain three study groups matched for age, BMI, waist circumference and blood pressure. The computer algorithm used to match patients was based on the minimum Euclidean distance rule. The necessary data were extracted from patients’ medical records stored online in a digital format. Because our unit is a tertiary center for adult patients with metabolic and hormonal disorders, height, weight, waist circumference and blood pressure had been routinely checked and recorded, even in otherwise healthy subjects. The study population was greater than the minimum sample size. An *a priori* power analysis showed that 26 or more women per group are required to reach a statistical power of 80% at α = 0.05. The participants were enrolled either in January or February (45 women: 16 in group A, 15 in group B and 14 in group C), or in July or August (47 women: 16 in group A, 16 in group B and 15 in group C) in order to limit the impact of seasonal confounds and seasonal fluctuations in the outcome variables.

Because severe hyperprolactinemia and pituitary tumors require specific treatment, macroprolactinomas, pituitary tumors co-secreting prolactin and other pituitary hormones and pseudoprolactinomas belonged to the exclusion criteria. The patients were also excluded if they met at least one of the following criteria: macroprolactinemia, other pituitary disorders, thyroid, parathyroid or adrenal disorders, diabetes, premature or early menopause, cardiovascular disease, autoimmune or inflammatory diseases, kidney or liver failure, malabsorption syndromes, other serious disorders, pregnancy or lactation, any pharmacotherapy (except for antipsychotic drugs) and poor patient compliance.

**Figure 1:**
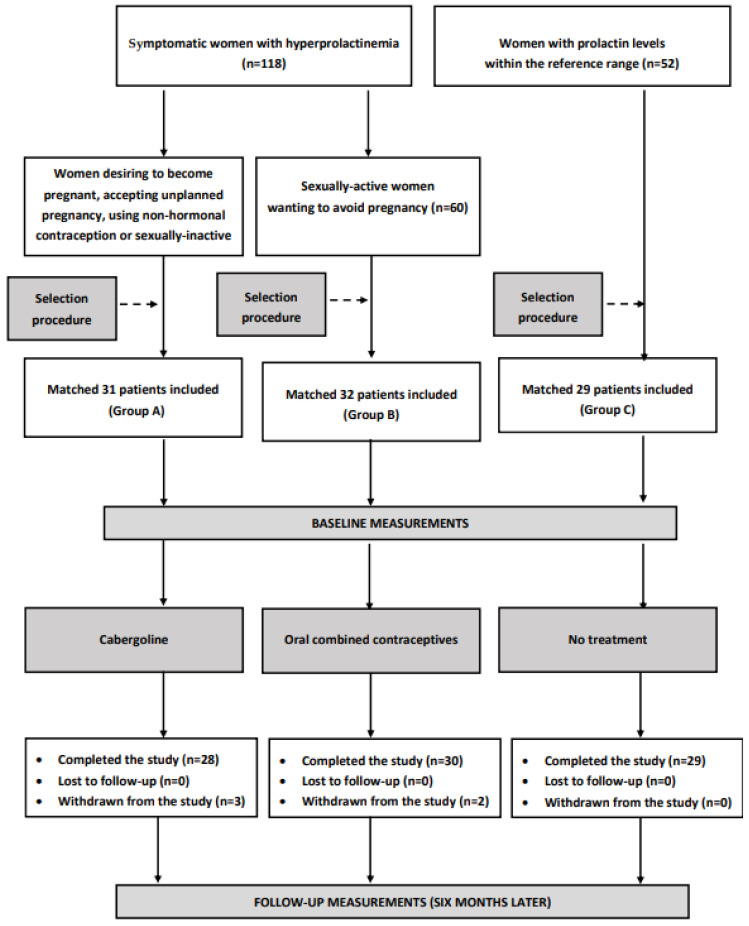
The flow of patients through the main study.

The flow of patients through the main study is depicted in Figure 1.

Because symptomatic patients with prolactin excess always require specific treatment, use of a placebo or leaving patients with elevated prolactin levels without treatment were consider unethical. To support or eliminate the impact of non-pharmacological treatment on the obtained results, we carried out a parallel analysis of 12 women, aged between 20 and 50, with iatrogenic prolactin excess, not participating in the main study. These women, meeting the inclusion criteria but declining pharmacotherapy of hyperprolactinemia (because of fear of psychosis exacerbation), were supervised by our research group in the same period of time as the remaining groups. The remaining 14 women, who had declined pharmacotherapy of hyperprolactinemia, were not interested in complying with the lifestyle intervention and/or in being supervised by our research group, and were not analyzed.

### 2.2. Study Design

Over the entire study period (six months), group A received cabergoline, group B was treated with oral contraceptive pills, while group C did not receive any treatment. Cabergoline was up-titrated from a starting dose of 0.25 mg once weekly in the first two weeks, to 0.25 twice weekly from the third week onwards. Group B received 30 μg of ethinyl estradiol and 150 μg of desogestrel. The pills were taken each day for 21 days followed by a 7-day rest, and these cycles were repeated without interruption throughout the study. All three groups of patients, as well as hyperprolactinemic women declining pharmacotherapy of prolactin excess followed the lifestyle modification (total fat intake < 30% of total energy intake, saturated fat intake < 7% of energy consumed, cholesterol intake < 200 mg per day, fiber intake ≥ 15 g per 1000 kcal, moderate to vigorous exercise for at least 30 min per day). In patients with drug-induced prolactin excess, no changes in dosage of antipsychotic drugs were allowed. Short-term (for less than seven days) use of other drugs was accepted only if such treatment was terminated at least four weeks before the end of the study. Drug adherence was measured every eight weeks by counting the number of residual tablets, and was considered satisfactory if the percentage of tablets returned was in the range from 0% to 10%. Adherence to non-pharmacological recommendations was assessed by analysis of individual dietary questionnaires and diaries in which the patients recorded all their activities.

### 2.3. Laboratory Assays

All laboratory assays were carried out in duplicate at baseline and on the last day of the intervention/observation period. Venous blood samples were drawn from antecubital vein, between 7.30 and 8.30 a.m., at least 12 h after the last meal and assessed by a person blinded to the treatments. Before venipuncture, all participants had been resting for at least 30 min in the seated position. Standard laboratory techniques were used to measure plasma levels of glucose, total cholesterol, low-density lipoprotein (LDL)-cholesterol, high-density lipoprotein (HDL)-cholesterol, triglycerides and uric acid, as well as urine levels of albumin and creatinine (Roche Diagnostics, Basel, Switzerland). Glycated hemoglobin was measured in whole blood samples using turbidimetric inhibition immunoassay on the Cobas Integra 800 analyzer (Roche Diagnostics, Mannheim, Germany). Plasma levels of insulin, prolactin and homocysteine were detected by direct chemiluminescence using acridinium ester technology (ADVIA Centaur XP Immunoassay System, Siemens Healthcare Diagnostics, Munich, Germany). Prolactin levels were assayed both before (total prolactin) and after (monomeric prolactin) polyethylene glycol precipitation. Macroprolactin content was calculated by subtracting monomeric prolactin from total prolactin. Circulating levels of hsCRP were measured by immunoassay with chemiluminescent detection (Immulite 2000XPi, Siemens Healthcare, Warsaw, Poland), while plasma fibrinogen was assessed using the Clauss method (BCS XP autoanalyzer, Siemens Healthcare, Warsaw, Poland). The homeostasis model assessment 1 of insulin resistance index (HOMA1-IR) was calculated as plasma glucose level (mg/dL) × insulin level (mU/L)/405. Urinary albumin-to-creatinine ratio (UACR) was calculated by dividing the value of urinary albumin by urinary creatinine.

### 2.4. Statistical Analysis

All variables were log-transformed in order to obtain a Gaussian-shaped distribution. Comparisons between the study groups and between percentage changes from baseline after adjustment for baseline values were carried out using either one-way analysis of variance followed by the *post-hoc* Bonferroni test (comparisons between three groups) or Student’s unpaired *t*-test (comparisons between two groups). The differences between follow-up and baseline values within the same study group were compared with the Student’s paired *t*-test. Dichotomized or nominal variables were compared using *χ*^2^ tests. Correlations between the investigated variables were assessed using Pearson’s r tests for two continuous variables; Phi coefficient for one continuous and one categorical variable; and Point-biserial for two categorical variables. The level of significance was set at *p* corrected for multiple testing below 0.05.

## 3. Results

At baseline, there were no statistically significant differences between the study groups in age, smoking habits, reasons for hyperprolactinemia, BMI, waist circumference, blood pressure, macroprolactin, total cholesterol and LDL-cholesterol. Duration of symptoms was similar in both groups of patients with elevated prolactin levels. Groups A and B differed from group C in plasma prolactin (total and monomeric), glucose, HOMA1-IR, glycated hemoglobin, HDL-cholesterol, triglycerides, uric acid, hsCRP, fibrinogen, homocysteine and UACR. There were no differences between groups A and B (Table 1 and Table 2).

In group A, three women prematurely terminated the study: two because of pregnancy and one owing to non-compliance with the study protocol. Cabergoline use was stopped after confirming pregnancies, and both pregnancies ended in deliveries of healthy infants. In group B, one patient experienced breast tenderness and vaginal itching, while another subject complained of nausea and abdominal pains. Both patients were withdrawn from the study. There were no dropouts in group C. Neither significant adverse effects nor any other complications were reported for the remaining 87 women (28 in group A, 30 in group B and 29 in group C) who completed the study protocol and were subjected to statistical analyses. All analyzed women complied with treatment and dietary recommendations and there were no between-group differences in physical activity. A *post-hoc* power calculation based on the primary outcome data and the given sample size showed that the study had sufficient statistical power (0.90).

Only cabergoline reduced total and monomeric prolactin levels, while in the remaining two groups there were no differences between follow-up and baseline concentrations of this hormone. At the end of the study, prolactin levels (both total and monomeric) within the reference range were observed in all patients from groups A and C but in no patient from group B. There were no differences between follow-up and baseline levels of macroprolactin. Cabergoline also decreased plasma glucose, HOMA1-IR, glycated hemoglobin, triglycerides, uric acid, hsCRP, fibrinogen, homocysteine and UACR, as well as increased HDL-cholesterol. Follow-up values of these parameters in group A did not differ from those observed in group C. Ethinyl estradiol plus desogestrel increased HOMA1-IR, triglycerides, hsCRP, fibrinogen and UACR. With the exception of macroprolactin, total cholesterol and LDL-cholesterol, follow-up values of all assessed parameters differed between group B and the remaining two groups (Table 2). Groups A and B differed in the percentage changes from baseline in total prolactin, monomeric prolactin, glucose, glycated hemoglobin, HDL-cholesterol, triglycerides, uric acid, hsCRP, fibrinogen, homocysteine and UACR (Table 3). There were no differences between follow-up and baseline values of BMI, waist circumference and blood pressure (data not shown).

In patients refusing cabergoline and oral contraceptives but complying with the lifestyle intervention program, total prolactin, glucose homeostasis markers, lipids, uric acid, hsCRP, fibrinogen and homocysteine remained at similar levels for the entire period of observation (Table 4).

Baseline prolactin levels positively correlated with baseline values of fasting glucose, HOMA1-IR, triglycerides, uric acid, hsCRP, fibrinogen, homocysteine and UACR (*r* values between 0.29 (*p* = 0.0446) and 0.48 (*p* < 0.0001) for total prolactin, and between 0.32 (*p* = 0.0356) and 0.52 (*p* < 0.0001) for monomeric prolactin), and inversely correlated with HDL-cholesterol (total prolactin: *r* = −0.38; *p* = 0.0012; monomeric prolactin: *r* = −0.40; *p* = 0.0008). In group A, there were correlations between the impact of cabergoline on prolactin levels and treatment-induced changes in fasting glucose, HOMA1-IR, glycated hemoglobin, HDL-cholesterol, triglycerides, uric acid, hsCRP, fibrinogen, homocysteine and UACR (*r* values between 0.30 (*p* = 0.0416) and 0.46 (*p* = 0.0001) for total prolactin, and between 0.34 (*p* = 0.0204) and 0.50 (*p* < 0.0001) for monomeric prolactin). In group B, the impact of treatment on fibrinogen and UACR inversely correlated with baseline levels of hsCRP (*r* = −0.35 (*p* = 0.0112) for fibrinogen; *r* = −0.37 (*p* = 0.0078) for UACR), and positively with the changes in hsCRP (*r* = 0.40 (*p* = 0.0008) for fibrinogen; *r* = 0.39 (*p* = 0.0010) for UACR). In the same study group, treatment-induced increase in hsCRP positively correlated with the impact of treatment on HOMA1-IR (*r* = 0.47; *p* < 0.0001), triglycerides (*r* = 0.41; *p* = 0.0008), fibrinogen (*r* = 0.37; *p* = 0.0086) and UACR (*r* = 0.34; *p* = 0.0285). The remaining correlations did not reach the level of significance.

## 4. Discussion

Higher values of most cardiometabolic risk factors assessed in the current study, correlating with baseline prolactin levels, indicate that even mild-to-moderate prolactin excess is associated with an increased risk of development of cardiovascular disease and carbohydrate disorders, and that this risk is proportional to the degree of hyperprolactinemia. Because the study population included women with prolactin excess of different origin: subjects with microprolactinoma, drug-induced prolactin excess, traumatic brain injury, empty sella syndrome and idiopathic hyperprolactinemia, the increased risk does not seem to depend on the underlying condition. The lack of patients with macroprolactinemia (resulting from the exclusion criteria), no between-group differences in macroprolactin content and the presence of correlations of the investigated cardiometabolic risk factors with monomeric prolactin but not with its high-molecular-weight forms indicate that our findings reflected increased circulating levels of monomeric prolactin. Owing to the selection procedure and strict inclusion and exclusion criteria, baseline differences between hyperprolactinemic and normoprolactinemic women cannot be also attributed to comorbidities or to other drugs used by the study population.

The treatment groups markedly differed in the impact on prolactin levels. Although administered in a relatively low dose, cabergoline normalized plasma levels of this hormone and in all cabergoline-treated women follow-up prolactin levels were within the reference range. Moreover, two cabergoline-treated women got pregnant and there were no cases of adverse effects in subjects receiving this agent. Thus, our findings seem to support the view that cabergoline treatment is an effective, safe and well-tolerated approach for hyperprolactinemia [13]. In turn, the impact of oral contraception on plasma was neutral, which suggests that 30 μg of ethinyl estradiol combined with 150 μg of desogestrel do not modulate secretory function of overactive gonadotropes. This finding is in contrast with previous observations indicating that oral contraceptive users were characterized by higher incidence of hyperprolactinemia than control subjects, and that the increase in prolactin levels was noted in 12–30% of patients receiving higher estrogen-containing oral contraceptives [26]. Another observation resulting from our study is that combined oral contraceptives do not affect macroprolactin content in women with baseline levels of high-molecular-weight prolactin within the reference range. Different effects on prolactin levels allow us to conclude that, from a hormonal point of view, combined oral contraceptive pills are inferior to cabergoline but do not seem to exacerbate existing hyperprolactinemia.

However, the most important finding of the current study are between-group differences in the impact on cardiometabolic risk factors. Cabergoline normalized plasma levels of all assessed factors, and this effect correlated with the impact on total and monomeric prolactin levels. Moreover, follow-up values of all these variables did not differ from those observed in the matched control women with normal prolactin levels. In turn, oral contraceptives worsened insulin sensitivity and increased triglycerides, hsCRP, fibrinogen and UACR, already impaired by the presence of prolactin excess. Our findings suggest that combined oral contraceptive pills may potentiate insulin resistance, low-grade systemic inflammation, procoagulant activity and early vascular damage, induced by hyperprolactinemia itself. Considering a predictive role of these biomarkers [27,28], they may facilitate subsequent development of cardiovascular and metabolic complications in hyperprolactinemic women.

Differences in cardiometabolic effects of cabergoline and oral contraception seem to reflect superiority of the former treatment option. Both treated groups of women with hyperprolactinemia did not differ in baseline characteristics. They were characterized by similar levels of total prolactin, monomeric prolactin and macroprolactin, similar reasons for hyperprolactinemia, similar duration of symptoms, and did not receive medications (with the except for antipsychotic agents taken by similar proportions of patients). The differences do not seem to be associated also with the impact of diet and physical activity. Over the entire study period, both groups of hyperprolactinemic women receiving pharmacotherapy adhered to the same non-pharmacological recommendations and did not differ in recommended physical activity. Moreover, there were no differences in plasma glucose, HOMA1-IR, lipids, uric acid, hsCRP, fibrinogen and homocysteine in women with iatrogenic hyperprolactinemia refusing cabergoline and hormonal contraception but complying with the lifestyle modification program for the same period of time (six months) as the main study population. In turn, similar baseline and follow-up levels of cardiometabolic risk factors in women declining pharmacotherapy of iatrogenic prolactin excess and in untreated women with normal prolactin levels argue against adaptive changes in response to prolactin excess. Certainly, it cannot be excluded that between-group differences in cardiometabolic effects of both treatment options may to some, albeit probably small, degree, be a consequence of imbalances in undetected comorbidities, unmeasured confounders (e.g., stress, sleep quality, or sexual activity), undetected side-effects (e.g., mild liver disease induced by ethinyl estradiol), and/or not analyzing the results of withdrawn subjects.

Based on the obtained results some conclusions may be drawn. Firstly, hyperprolactinemia may increase cardiometabolic risk proportionally to the degree of prolactin excess. Correlations between prolactin levels and the assessed biomarkers suggest that this risk may be greatest in individuals with severe hyperprolactinemia, not included in the present study. Secondly, clinical improvement cannot be regarded as the only treatment goal in women with prolactin excess. Thirdly, benefits of cabergoline treatment seem to go beyond normalizing prolactin levels and the clinical improvement. The drug may prevent or slow down the development of cardiovascular disease, type 2 diabetes and other insulin-resistant states. Fourthly, if the treatment is started in subjects without irreversible changes in the vascular system and glucose homeostasis, the risk of their development in cabergoline-treated subjects may be similar to that observed in their healthy peers. Furthermore, combined oral contraceptive pills should be avoided as first choice treatment of prolactin excess in patients with symptomatic hyperprolactinemia if their cardiometabolic risk is increased (owing to comorbidities or genetic predisposition). Lastly, high cardiometabolic risk women with hyperprolactinemia who, because of resistance or poor tolerance, cannot be treated with dopaminergic agents may gain some benefits in the case when estrogen-progestogen preparations are administered together with other agents known to reduce the risk of cardiovascular or metabolic disorders.

Another interesting observation resulting from our study is that unfavorable cardiometabolic effects of oral contraceptives may depend on the degree of low-grade inflammation and on their effect on systemic inflammation. In line with this explanation, the impact of oral contraceptive pills on fibrinogen and UACR inversely correlated with baseline levels of hsCRP, a well-documented marker of systemic inflammation [29], as well as positively with treatment-induced changes in hsCRP. Moreover, proinflammatory cytokines and other mediators of inflammation were found to stimulate fibrinogen production [30] and urinary albumin loss [31]. This finding suggests that hyperprolactinemic women with high levels of this protein may be poor candidates for monotherapy with oral contraceptive pills. However, their unfavorable cardiometabolic properties may be reversed in case of concomitant therapy with drugs found to reduce systemic inflammation: cyclooxygenase inhibitors, statins, ezetimibe, fenofibrate, angiotensin-converting enzyme inhibitors, some sartans or antioxidants [32].

In the current study, low-grade inflammation was also associated with insulin resistance. In patients taking oral contraceptive pills, the correlation between hsCRP and HOMA1-IR was stronger than correlations between hsCRP and fibrinogen, as well as between hsCRP and UACR. This finding suggests that the impact of combined oral contraceptive pills on low-grade systemic inflammation may partially determine their effect on insulin receptor action. It seems that low-grade systemic inflammation and combined oral contraceptive pills may interact at the level of GLUT4, mediating the rate-limiting glucose cellular uptake in adipocytes and muscle cells, and thus playing an important role in insulin-responsive glucose metabolism [33]. In line with this explanation, conjugated equine estrogens administered together with medroxyprogesterone acetate down-regulated GLUT4 expression [34], and a similar effect on translocation and membrane expression of this transporter was induced by proinflammatory cytokines (interleukin-1ß, interferon-γ and tumor necrosis factor-α) [35,36,37], the production of which is stimulated by prolactin excess [38]. Interestingly, apart from lowering prolactin levels, cabergoline may improve glucose homeostasis also at the level of GLUT4 expression, which was found to be up-regulated by dopaminergic agents [39]. However, in opposition to previous findings [15,16,17,18], despite normalizing prolactin levels, cabergoline had a neutral effect on BMI, waist circumference and blood pressure. This discrepancy may have resulted from low baseline values of all these parameters in the participants of the current study and from the relatively short treatment duration.

Carbergoline treatment increases the risk of cardiac valve disease, and this effect is mediated by the 5-HT_2B_ agonist activity on the serotoninergic receptors expressed on cardiac valvular fibroblasts. The risk of valvular dysfunction is dose-dependent. Valvulopathy was observed almost exclusively in patients treated, because of Parkinson’s disease, with at least 3 mg of cabergoline daily and/or if a cumulative dose exceeded some threshold value (between 2.6 and 6.7 g) [40,41]. In case of low doses, recommended to most patients with prolactin excess and used in the current study, even long-term treatment does not seem to predispose to the development of structural cardiac complications [41,42]. In line with this view, no our patient developed cardiac valve disease. Thus, low doses of cabergoline seem to be a safe treatment of hyperprolactinemic patients, and cardiometabolic benefits resulting from this treatment do not seem to be counterbalanced by an increased risk of valvular heart disease. However, because patients with structural heart disorders did not participate in the study, our findings cannot be generalized to all patients receiving this agent.

## 5. Study Limitations

Interpretations stemming from our findings should be considered in light of some study limitations. The most important one is the small sample size. Strict inclusion and exclusion criteria minimized the possible impact of comorbidities or comedications on the obtained results, but also decreased the number of enrolled women. Moreover, patient recruitment was prematurely terminated by the outbreak of the COVID-19 pandemic. Thus, although a *post-hoc* power analysis showed that the sample size was sufficiently powered for a designated endpoint, our findings should be interpreted as hypothesis-generating rather than definitive conclusions. The obtained results might have been also influenced by latent confounders and selection bias because the study was non-randomized and, for ethical reasons, did not include a group of placebo-treated women. Treatment-induced changes in surrogate markers cannot be easily translated to hard endpoints. It is uncertain whether the effect of hormonal contraception is the same in women receiving ethinyl estradiol and desogestrel at other doses than used in the current study, or receiving other estrogen-progestogen combinations. Because the study population included a heterogenous group of women with prolactin excess, it cannot be totally excluded that cardiometabolic effects of both treatment options depend on the reason for hyperprolactinemia. The study protocol minimized the impact of random diurnal, seasonal and analytical variations in the outcome variables, reducing but not eliminating the regression-toward-the-mean effect [43]. Lastly, although all *p*-values were adjusted for multiple testing (because of the large number of comparisons), the risk of obtaining a false positive result (type 1 error) cannot be completely ruled out.

## 6. Conclusions

Hyperprolactinemic women differed from normoprolactinemic ones in glucose homeostasis markers, HDL-cholesterol, triglycerides, uric acid, hsCRP, fibrinogen, homocysteine and UACR. Beyond decreasing prolactin levels, cabergoline treatment improved most cardiometabolic risk factors assessed in the current study, while ethinyl estradiol/desogestrel combination therapy potentiated the unfavorable effect of prolactin excess on insulin sensitivity, triglycerides, hsCRP, fibrinogen and UACR. The obtained results suggest that cabergoline is superior to oral combined contraceptive pills in women when it comes to affecting cardiometabolic risk, and that monotherapy with oral combined contraceptives should be avoided if mild-to-moderate hyperprolactinemia coexists with the presence of other cardiometabolic risk factors. Due to the preliminary nature of the current research, the obtained results should be confirmed in large randomized clinical trials with longer follow-up.

## Figures and Tables

**Table 1:** Baseline characteristics of patients.

Variable	Group A	Group B	Group C
**Number** (*n*)	28	30	29
**Age** (years)	34 ± 7	35 ± 8	35 ± 7
**Smokers** (%)**/Number of cigarettes a day** (*n*)**/Duration of smoking** (months)	25/10 ± 6/110 ± 32	23/9 ± 6/114 ± 28	28/9 ± 5/118 ± 35
**Reasons for prolactin excess** (%)**: microprolactinoma/drug-induced hyperprolactinemia/traumatic brain injury/empty sella syndrome/idiopathic**	14/36/25/18/7	13/40/23/17/7	17/41/24/14/4
**Duration of hyperprolactinemia symptoms** (months)	8 ± 5	7 ± 6	-
**BMI** (kg/m^2^)	24.4 ± 4.0	24.2 ± 3.5	23.7 ± 4.2
**Waist circumference** (cm)	84 ± 8	84 ± 7	82 ± 8
**Systolic blood pressure** (mmHg)	131 ± 12	129 ± 14	127 ± 14
**Diastolic blood pressure** (mmHg)	83 ± 6	82 ± 5	81 ± 6

Group A: hyperprolactinemic women treated with cabergoline. Group B: hyperprolactinemic women treated with combined oral contraceptive pills Group C: Untreated women with prolactin levels within the reference range. Unless otherwise stated, the data are presented as the mean ± standard deviation. Abbreviations: BMI, body mass index.

**Table 2:** The effect of cabergoline and combined oral contraceptive pills on the investigated variables in the study population.

Variable	Group A	Group B	Group C
**Total prolactin** (ng/mL)			
*Baseline*	57.0 ± 11.0 ^#^	55.5 ± 12.0 ^#^	15.0 ± 7.1
*Follow-up*	15.2 ± 7.0 ^$^	56.8 ± 10.6 *^#^	14.6 ± 7.5
**Monomeric prolactin** (ng/mL)			
*Baseline*	54.2 ± 10.8 ^#^	52.5 ± 11.9 ^#^	12.4 ± 6.4
*Follow-up*	12.3 ± 6.4 ^$^	54.1 ± 10.5 *^#^	12.1 ± 6.8
**Macroprolactin** (ng/mL)			
*Baseline*	2.8 ± 1.2	3.0 ± 1.4	2.6 ± 1.1
*Follow-up*	2.9 ± 1.0	2.7 ± 1.2	2.5 ± 1.0
**Glucose** (mg/dL)			
*Baseline*	95 ± 12 ^#^	94 ± 13 ^#^	86 ± 12
*Follow-up*	87 ± 10 ^$^	95 ± 11 *^#^	85 ± 11
**HOMA1-IR**			
*Baseline*	3.0 ± 0.8 ^#^	2.9 ± 0.8 ^#^	1.5 ± 0.6
*Follow-up*	1.7 ± 0.5 ^$^	3.5 ± 1.1 *^#$^	1.6 ± 0.5
**Glycated hemoglobin** (%)			
*Baseline*	5.5 ± 0.2 ^#^	5.5 ± 0.3 ^#^	5.2 ± 0.3
*Follow-up*	5.2 ± 0.2 ^$^	5.5 ± 0.2 *^#^	5.2 ± 0.2
**Total cholesterol** (mg/dL)			
*Baseline*	201 ± 50	203 ± 42	194 ± 46
*Follow-up*	194 ± 46	205 ± 43	195 ± 47
**HDL-cholesterol** (mg/dL)			
*Baseline*	48 ± 9 ^#^	48 ± 8 ^#^	56 ± 10
*Follow-up*	55 ± 10 ^$^	46 ± 8 *^#^	55 ± 11
**LDL-cholesterol** (mg/dL)			
*Baseline*	118 ± 32	120 ± 26	115 ± 34
*Follow-up*	110 ± 29	121 ± 30	112 ± 28
**Triglycerides** (mg/dL)			
*Baseline*	152 ± 48 ^#^	148 ± 42 ^#^	118 ± 32
*Follow-up*	120 ± 35 ^$^	174 ± 47 *^#$^	121 ± 38
**Uric acid** (mg/dL)			
*Baseline*	4.8 ± 1.3 ^#^	4.9 ± 1.2 ^#^	4.2 ± 1.1
*Follow-up*	4.0 ± 1.0 ^$^	5.0 ± 1.5 *^#^	4.4 ± 1.3
**hsCRP** (mg/L)			
*Baselin*	2.6 ± 1.0 ^#^	2.8 ± 0.9 ^#^	1.2 ± 0.3
*Follow-up*	1.2 ± 0.4 ^$^	3.6 ± 1.0 *^#$^	1.1 ± 0.4
**Fibrinogen** (mg/dL)			
*Baseline*	372 ± 75 ^#^	358 ± 95 ^#^	288 ± 70
*Follow-up*	302 ± 83 ^$^	455 ± 105 *^#$^	294 ± 64
**Homocysteine** (μmol/L)			
*Baseline*	25.6 ± 10.1 ^#^	24.2 ± 10.2 ^#^	11.2 ± 4.3
*Follow-up*	12.7 ± 5.2 ^$^	26.5 ± 11.4 *^#^	11.6 ± 5.0
**UACR** (mg/g)			
*Baseline*	31.5 ± 8.3 ^#^	30.4 ± 8.8 ^#^	8.5 ± 2.3
*Follow-up*	10.0 ± 4.8 ^$^	36.8 ± 9.2 *^#$^	9.0 ± 4.2

Group A: hyperprolactinemic women treated with cabergoline. Group B: hyperprolactinemic women treated with combined oral contraceptive pills. Group C: Untreated women with prolactin levels within the reference range. The data are presented as the mean ± standard deviation. * *p* < 0.05 vs. group A. ^#^ *p* < 0.05 vs. group C. ^$^ *p* < 0.05 vs. baseline value. Reference values for young women: total prolactin: 5.0–29.0 ng/mL monomeric prolactin: 4.0–26.0 ng/mL; macroprolactin: 2.0–4.0 ng/mL; glucose: 70–99 mg/dL, HOMA1-IR: <2.0; glycated hemoglobin: <5.6%; total cholesterol: <200 mg/dL; HDL-cholesterol ≥ 50 mg/dL; LDL-cholesterol: <115 mg/dL; triglycerides: <150 mg/dL; uric acid: 3.5–8.5 mg/dL; hsCRP: <1.0 mg/L; fibrinogen: 200–400 mg/dL; homocysteine: 4–14 μmol/L; UACR: <30 mg/g. Abbreviations: HDL, high-density lipoprotein; HOMA1-IR, the homeostatic model assessment 1 of insulin resistance ratio; hsCRP, high-sensitivity C-reactive protein; LDL, low-density lipoprotein; UACR, urinary albumin-to-creatinine ratio.

**Table 3:** Percentage changes from baseline in the investigated variables in hyperprolactinemic women receiving cabergoline or oral combined contraceptives.

Variable	Group A	Group B
**Δ Total prolactin**	−73 ± 12	2 ± 8
**Δ Monomeric prolactin**	−77 ± 10 *	3 ± 7
**Δ Macroprolactin**	−9 ± 10	−8 ± 9
**Δ Glucose**	−8 ± 5 *	1 ± 2
**Δ HOMA1-IR**	−43 ± 12 *	21 ± 8
**Δ Glycated hemoglobin**	−5 ± 3 *	0 ± 5
**Δ Total cholesterol**	−4 ± 8	1 ± 7
**Δ HDL-cholesterol**	15 ± 6 *	−4 ± 6
**Δ LDL-cholesterol**	−7 ± 12	1 ± 8
**Δ Triglycerides**	−21 ± 12 *	18 ± 10
**Δ Uric acid**	−17 ± 10 *	2 ± 12
**Δ hsCRP**	−54 ± 18 *	29 ± 14
**Δ Fibrinogen**	−19 ± 12 *	27 ± 15
**Δ Homocysteine**	−50 ± 18 *	10 ± 15
**Δ UACR**	−68 ± 20 *	21 ± 10

Group A: hyperprolactinemic women treated with cabergoline. Group B: hyperprolactinemic women treated with combined oral contraceptive pills. The data are presented as the mean ± standard deviation. * *p* < 0.05 vs. group B. Δ–difference between follow-up and baseline value. Abbreviations: HDL, high-density lipoprotein; HOMA1-IR, the homeostatic model assessment 1 of insulin resistance ratio; hsCRP, high-sensitivity C-reactive protein; LDL, low-density lipoprotein; UACR, urinary albumin-to-creatinine ratio.

**Table 4:** The impact of complying with the lifestyle modification program on the investigated variables in patients with iatrogenic hyperprolactinemia declining pharmacotherapy of prolactin excess.

Variable	Baseline	Follow-Up (Six Months Later)
**Total prolactin** (ng/mL)	57.8 ± 159	58.9 ± 17.8
**Glucose** (mg/dL)	96 ± 13	95 ± 12
**HOMA1-IR**	3.1 ± 1.1	2.9 ± 1.0
**Total cholesterol** (mg/dL)	204 ± 52	208 ± 49
**HDL-cholesterol** (mg/dL)	48 ± 12	50 ± 14
**LDL-cholesterol** (mg/dL)	122 ± 40	125 ± 38
**Triglycerides** (mg/dL)	160 ± 55	148 ± 52
**Uric acid** (mg/dL)	5.0 ± 2.0	4.8 ± 1.8
**hsCRP** (mg/L)	2.8 ± 1.2	2.8 ± 1.4
**Fibrinogen** (mg/dL)	380 ± 140	406 ± 165
**Homocysteine** (μmol/L)	23.8 ± 12.4	23.1 ± 11.8

The data are presented as the mean ± standard deviation. Macroprolactin, monomeric prolactin, glycated hemoglobin and urinary albumin-to-creatinine ratio were not determined in this population. Abbreviations: HDL, high-density lipoprotein; HOMA1-IR, the homeostatic model assessment 1 of insulin resistance ratio; hsCRP, high-sensitivity C-reactive protein; LDL, low-density lipoprotein.

## Data Availability

The data that support the findings of this study are available from the corresponding author upon reasonable request.

## Abbreviations

BMI—the body mass index; HDL—high-density lipoprotein; HOMA1-IR—the homeostatic model assessment 1 of insulin resistance ratio; hsCRP—high-sensitivity C-reactive protein; LDL—low-density lipoprotein; UACR—urinary albumin-to-creatinine ratio.
